# Case report: Differential diagnosis of highly amplified anti-CD5 CAR T cells and relapsed lymphoma cells in a patient with refractory ALK positive anaplastic large cell lymphoma

**DOI:** 10.3389/fimmu.2023.1280007

**Published:** 2023-12-08

**Authors:** Wei Mu, Meilan Zhang, Guang Hu, Yunfeng Han, Xia Mao, Caixia Chen, Kefeng Shen, Zhenyu Dai, Xiaojian Zhu, Xiaoxi Zhou, Liang Huang, Qilin Ao, Min Xiao

**Affiliations:** ^1^ Department of Hematology, Tongji Hospital, Tongji Medical College, Huazhong University of Science and Technology, Wuhan, Hubei, China; ^2^ Nanjing IASO Biotherapeutics Ltd., Nanjing, China; ^3^ Department of Nuclear Medicine, Tongji Hospital, Tongji Medical College, Huazhong University of Science and Technology, Wuhan, China; ^4^ Institute of Pathology, School of Basic Medical Science, Tongji Medical College, Huazhong University of Science and Technology, Wuhan, China; ^5^ Department of Pathology, Tongji Hospital, Tongji Medical College, Huazhong University of Science and Technology, Wuhan, China

**Keywords:** ALK-positive anaplastic large cell lymphoma, cell therapy, chimeric antigen receptor T cells, CD5 antigen, laboratory based differential diagnosis

## Abstract

**Background:**

Anaplastic Large Cell Lymphoma (ALCL) is one of the most common subtypes of T-cell lymphoma. Among these, refractory and relapsed (r/r) ALK positive ALCL lacks effective therapies. The chimeric antigen receptor-modified T (CAR-T) cell therapy holds great promise as a therapeutic strategy for this disease. However, it is not known yet whether anti-CD5 CAR-T cells are sufficient for the definitive treatment of relapsed ALK^+^ ALCL, nor the role of accurate laboratory-based diagnoses during CAR-T treatment.

**Case presentation:**

The adolescent patient received autologous T cells containing sequences encoding V_H_ domains specific to CD5. Following the infusion, there was an increase in both the copy number and proportion of CAR-T cells in peripheral blood. IL-6 and ferritin levels in the patient exhibited significant fluctuations, with increases of 13 and 70 folds respectively, compared to baseline after the treatment. Additionally, adverse effects were observed, including grade 4 rash, grade 1 headache, nausea, and neck-pain. Surprisingly, a relapsed disease phenotype was identified based on the results of PET/CT and histopathological analysis of the inguinal lymph node biopsy. After conducting a thorough diagnostic assessment, which included flow cytometry, next-generation sequencing (NGS), examination of immune-related gene rearrangements, and analysis of the immune repertoire of T-cell receptors (TCR), we conclusively determined that the hyperplastic T cells identified in the lymph node were the result of an expansion of CAR-T cells. Ultimately, the patient has attained complete remission (CR) and has sustained a disease-free survival state for 815 days as of the cutoff date on August 30, 2023.

**Conclusion:**

Taken together, the results demonstrate that anti-CD5 CAR-T cells can induce a clinical response in r/r ALK^+^ ALCL patient. Furthermore, this case underscores the importance of utilizing advanced technologies with high sensitivity and accuracy for biological detection in clinical laboratory diagnosis and prognosis in CAR-T cell treatment.

**Trial registration number:**

NCT04767308.

## Introduction

ALK^+^ ALCL is classified as a type of peripheral T cell lymphoma by the World Health Organization. It has a high occurrence among children and young adults, with a male predominance, accounting for 10-15% of pediatric and adolescent non-hodgkin lymphoma (NHL) and approximately 3% of adolescent NHL. The disease is commonly found in an advanced stage, affecting both nodal and various extra nodal sites, including the skin, soft tissue and lungs (1, 2). Current treatment strategies utilizing multiagent chemotherapy achieve an event free survival (EFS) rate of approximately 75% in children with ALK^+^ ALCL. While most patients have a favorable clinical course (3), a subset of them exhibit a more aggressive and refractory presentation (4). Therefore, it is a need to develop new effective therapeutic strategies for patients with advanced or refractory ALCL.

Chimeric antigen receptor modified T cells have shown promising efficacy in treating r/r B cell malignances (5, 6). However, our understanding of the response of CAR-T cell therapy in patients with T cell leukemia and lymphoma is still in its early stages. CD5 is a characteristic surface marker expressed in the majority of T-cell malignancies, including acute T lymphocytic leukemia (T-ALL), T-cell lymphoma, and some B-cell lymphomas, and is notably absent in hematopoietic stem cells and other non-hematopoietic cells (7). We previously showed that CD5 targeting CAR-T cells significantly suppressed the growth of T cell malignances both *in vitro* and *in vivo* (8), suggesting a promising clinical potential for anti-CD5 CAR-T product in treating the disease.

The emergence of new immunotherapy technologies has presented significant challenges to laboratory diagnosis, which is crucial for the diagnosis, prognosis, efficacy evaluation, and recurrence prediction of tumors (9). Generally, laboratory diagnoses encompass cell morphology analysis, flow cytometry immunotyping, cytogenetics, and molecular genetics. Among these, flow cytometry and droplet digital PCR (ddPCR) method have been widely employed to assess CAR-T cell proliferation and persistence *in vivo* (10). However, distinguishing between therapeutic, normal and neoplastic T cells poses a challenge, particularly in the context of T cell malignancy treatment (9). Therefore, new technologies and methods like NGS, immune repertoire, and gene rearrangement studies, which offer superior sensitivity in evaluating disease progression and marginal residual disease following CAR-T cell infusion, are needed.

In this study, an ALK^+^ ALCL patient with multiple lines of chemotherapy failures received an infusion of anti-CD5 CAR-T cells. The patient achieved CR with manageable adverse effects. Although T lymphocyte hyperplastic lesions were observed in the inguinal lymph nodes approximately 3 months after CAR-T cell infusion, further investigation confirmed that it was CAR-T cell proliferation rather than a relapse of ALCL. These results suggest that anti-CD5 CAR-T cells could serve as a novel therapeutic approach for the treatment of advanced or relapsed ALK^+^ ALCL. Furthermore, the utilization of new molecular biology-based diagnostic methods is crucial for accurately assessing disease status following CAR-T cell therapy.

## Case presentation

### The medical history

In August 2018, a 17-year-old male patient of Han ethnic group presented to local hospital with left cervical mass. He was diagnosed as being in the high-risk group with international prognostic index (IPI) score 4. The diagnosis indicated IVa stage ALK positive ALCL with recurrence after chemotherapy. Bone marrow smear analysis revealed hyperplastic and active bone marrow cells, with no significant increase in lymphocytes observed. Additionally, flow cytometry analysis did not detect any notable immunophenotypically abnormal lymphocytes, which revealed that the bone marrow was not infiltrated. Immunohistochemical staining of the cervical lymph node showed positive results for VIM (+), EMA (+), ALK (++), CD30 (++), CD4 (++), CD5 (++), CD2 (+), CD8 (partially+), CD15 (partially+), BCL-6 (partially+), MUM-1 (partially+), Ki67 (80%+), and negative results for CD3, CK, CK8/18, P63, TdT, MPO, CD20, CD79a, CD10, PAX-5, BCL-2 and HMB45. The patient received first-line chemotherapy with CHOPE (Isocyclophosphamide, Doxorubicin liposomes, Vincristine, Dexamethasone and Etoposide) for one cycle and achieved partial remission (PR). Subsequently, the patient underwent three cycles of chemotherapy with BFM-90-A (Dexamethasone, Cyclophosphamide, Vindesin, Methotrexate, Cytarabine and Etoposide) and BFM-90-B (Cyclophosphamide, DEX, VCR, Methotrexate, doxorubicin liposomes) respectively, resulting in CR as indicated by PET-CT. Nonetheless, in February 2021, the disease progressed, presenting with left abdominal distension and pain for one week, leading to a diagnosis of stage IVA ALK^+^ ALCL relapse. After receiving one cycle of Gemcitabine, Oxaliplatin and Vebutoximab, the patient achieved PR.

### Autologous anti-CD5 CAR-T cell therapy

**Figure 1:**
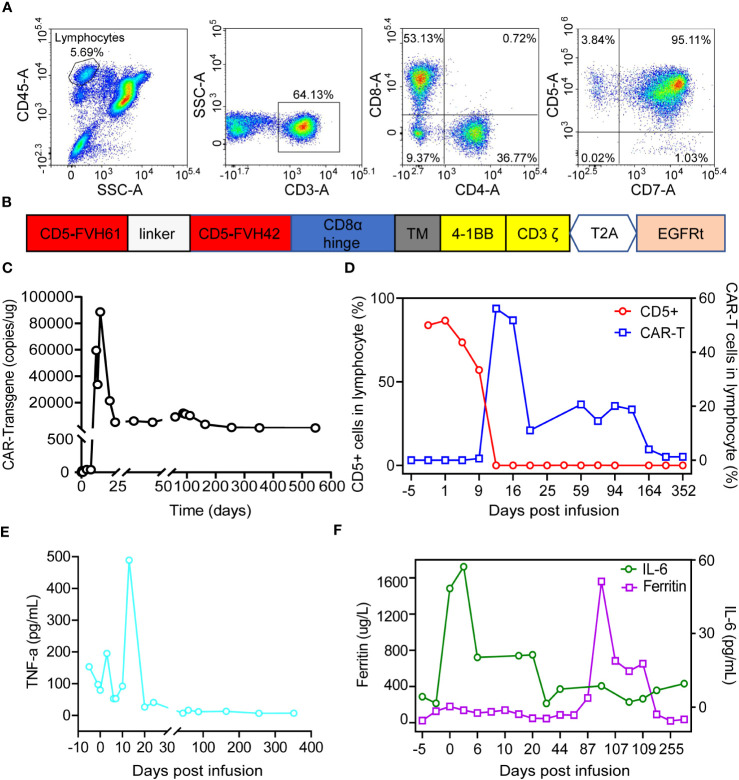
Characteristics of CD5 expression on normal lymphocytes in bone marrow at diagnosis and kinetics of CAR-T cells and inflammatory cytokines post CAR-T cells infusion. **(A)** Evaluation of CD5 antigen expression in normal lymphocytes of bone marrow before CAR-T cell treatment. **(B)** Structure of anti-CD5 CAR molecular. **(C)** Kinetic of CAR gene *in vivo* by using ddPCR. **(D)** Kinetic of CAR-T cells and CD5 positive cells *in vivo* by using flowcytometry. **(E)** Tumor necrosis factor α dynamics post CAR-T cells infusion. **(F)** Levels of IL-6 and ferritin in serum after CAR-T cell treatment.

The patient was referred to our hospital in March 2021 for further treatment and requested immunotherapy. Upon confirmation, CT scans revealed measurable nodules in the retroperitoneum, mesentery, and right pelvic wall. Expression of the CD5 antigen on the lymphocytes of bone marrow was measured by flow cytometry, which showed that 98% of CD3 positive cells expressed CD5 antigen (**Figure 1A**). In May 2021, the patient was accepted to participate in a clinical trial of anti-CD5 CAR-T cell therapy. The patient provided informed consent before undergoing apheresis.

The patient received a standard dose of Fludarabine, 50 mg/m^2^ and Cyclophosphamide, 500 mg/m^2^ (FC) regimen from day -4 to day -1 as lymphodepleting chemotherapy. Autologous anti-CD5 CAR-T cells were infused on June 12, 2021 (day 0) at a dose of 1.0 x 10^6/kg. The CAR structure (**Figure 1B**), as well as the manufacturing and preparation of CAR-T cells, were described previously (8). The amplification of CAR-T cells in peripheral blood was detected by ddPCR as reported (11). In **Figure 1C**, the copy number of the CAR-T product was 23 copies/μg DNA on day 0 and reached its peak level (88623 copies/μg DNA) on day 14. Flow cytometry was also conducted in the analysis of CAR-T cells and CD5 positive targeted cells during treatment. The proportion of CAR-T cells reached a peak level of 56.17% in lymphocyte cells and 62.88% in CD3^+^ T cells, while the proportion of CD5^+^ target cells in lymphocytes began to decrease on day 1 and reached to 0.00% on day 11 (**Figure 1D**). The absolute number of white blood cells, platelets, and hemoglobin level fluctuated within the normal range (**Supplemental Figure 1**). These results demonstrate that CAR-T cells but not for CD3^+^CD5^+^ T cells can be effectively expanded in patients, and the routine blood indicators including white cell, platelet and hemoglobin were basically normal.

The plasma level of cytokines, including IL-1β, IL-2R, IL-6, IL-8, IL-10, tumor necrosis factor (TNF-α), and ferritin, were measured using an electrochemiluminescence assay during anti-CD5 CAR-T cell treatment. TNF-α began to increase on day 1, reached its peak level (489 pg/mL) on day 13, and gradually decreased thereafter (**Figure 1E**). IL-8 exhibited a similar pattern to that of TNF-α. IL-2R remained at higher level throughout the treatment period, while IL-1β and IL-10 were nearly undetectable. The peak level of IL-6 was reached on day 3. Ferritin levels remained elevated throughout the treatment, with the highest level recorded on day 105 (**Figure 1F**).

The adverse events (AE) were evaluated according to the Common Terminology Criteria for Adverse Events (V.5.0). On day +7, the patient met the criteria for CRS grade 1, with the highest recorded body temperature being 38°C. On day +10, CRS subsided but was succeeded by a grade 1 CAR-T cell relevant encephalopathy syndrome (CRES), which persisted for 5 days. Other CAR-T related adverse events, such as neck pain, skin rash, headache, nausea, diarrhea, and abdominal pain, subsequently emerged. Throughout this period, methylprednisolone sodium succinate and cetuximab were administered to alleviate these symptoms.

### Hyperplastic T cells in inguinal lymph node were determined to be expanded CAR-T cells

**Figure 2:**
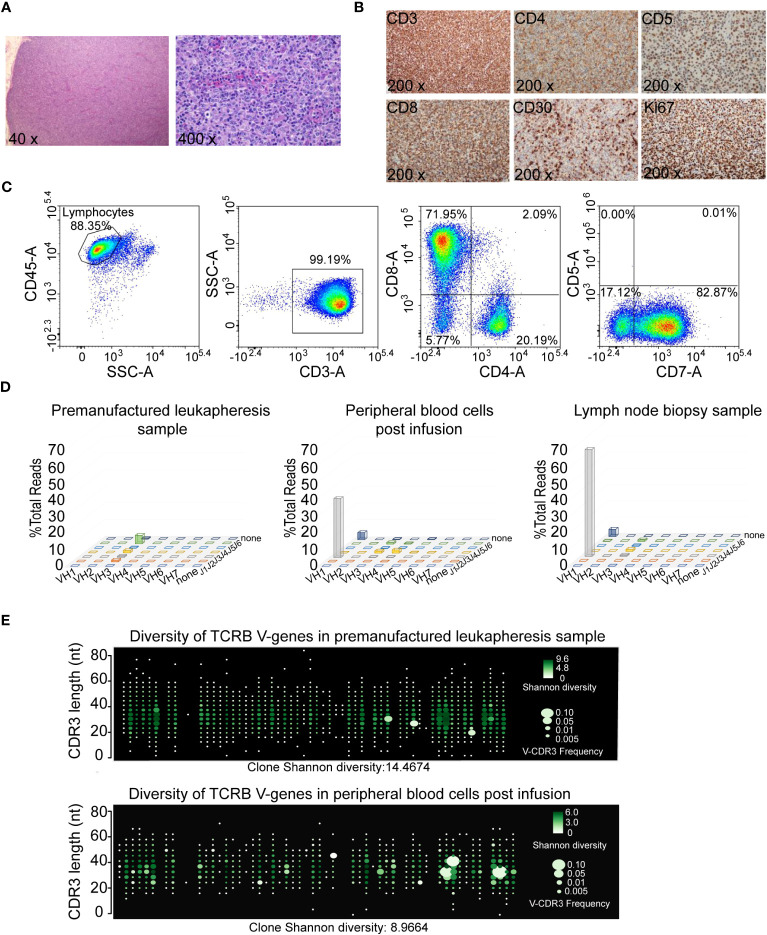
Hyperplastic T cells in inguinal lymph node turned out to be expanded CAR-T cells. **(A)** Histopathological microphotograph (hematoxylin and eosin, 40x and 400x). Normal structure of lymph node disappeared, and no lymph sinus and lymph follicles were found. The hyperplastic T cells are large in size, rich in cytoplasm, vacuolated nuclei with obvious nucleoli, and have a certain degree of atypia. **(B)** Immunohistochemical analysis of cervical lymph node biopsy for CD3, CD4, CD5, CD8, CD30 and Ki67 expression. **(C)** Immunophenotypic analysis of the inguinal lymph node biopsy. **(D)** Lymohotrack IGH assays in premanufactured leukapheresis sample, peripheral blood cells (59 days post CAR-T cells infusion) and lymph node biopsy sample detected by next generation sequencing. **(E)** TCRB V-gene usage and Shannon Diversity for peripheral blood cells (68 days post CAR-T cells infusion) and premanufactured leukapheresis sample were detected by immune repertoire assay.

Approximately three months after CAR-T cell infusion, the patient presented with multiple painless enlarged lymph nodes in the bilateral neck and groin on September 8, 2021, as evidenced by PET. Hematoxylin-Eosin (HE) staining of cervical lymph node biopsy showed that the loss of normal structure of lymph node architecture, with the absence of lymph sinuses or follicles. The hyperplastic T cells displayed enlarged size, abundant cytoplasm, vacuolated nuclei with prominent nucleoli, and exhibited a certain degree of atypia, consistent with features typically associated with refractory ALCL (**Figure 2A**). Immunohistochemical (IHC) staining of cervical lymph nodes showed diffuse positivity for CD2, CD3, CD7, CD8, TIA1, CD30 (with scattered positivity), Ki67 (80% labeling index), and negativity for CD4, CD5, CD20, EBER, and ALK (**Figure 2B** and **Supplemental Figure 2A**). This immunophenotype is inconsistent with the previously observed ALK-positive ALCL profile.

To discern whether the hyperplastic T lymphocytes observed in inguinal lymph node were a result of CAR-T cell expansion or aberrant tumor cell presence, a series of analyses were performed. Flow cytometry results from the inguinal lymph node biopsy showed that abnormal lymphocytes were negative for CD5 expression (**Figure 2C**), indicating the proficiency of anti-CD5 CAR-T cells in eliminating CD5 positive cells. Genomic DNA was extracted from the inguinal lymph node biopsy sample, followed by the establishment of a library using a multiple amplicon method. High throughput deep sequencing and Sanger sequencing were subsequently performed. This comprehensive analysis identified a total of 157 lymphoma related hot genes, all showing no mutations. Rearrangements of *IGHA*, *IGHB* and *TCR* genes were assessed in the lymph node biopsy sample, peripheral blood cells post infusion, and premanufactured leukapheresis sample using next generation sequencing, and these findings were further corroborated by Sanger sequencing. Clonal rearrangement *IGHA* and *IGHB* genes were evident in both the lymph node biopsy and peripheral blood cells, but not in the premanufactured leukapheresis sample (**Figure 2D**). Specific primers targeting *IGHA*, *IGHB* and *TCR* gene locus were designed for Sanger sequencing. Results revealed that the rearrangement fragment of *IGHA* and *IGHB* were attributed to the CAR sequence (**Supplemental Figure 2B**), and no monoclonal rearrangement was found in the *TCR* gene region. To further ascertain the diversity of TCR clones in peripheral blood cells post CAR-T cells infusion and the premanufactured leukapheresis sample, immune repertoire assays were conducted. The Shannon diversity index was determined to be 8.97, with the identification of 6474 T cell clones in the patient’s peripheral blood post infusion. In contrast, in the premanufactured leukapheresis, the Shannon diversity and clone numbers were calculated to be 14.47 and 53808, respectively (**Figure 2E** and **Supplemental Figure 2C**). These results collectively suggest that there is no discernible preferential expansion of abnormal T cell clones occurring.

Furthermore, the patient underwent follow-up clinical monitoring after the CAR-T treatment. On February 22, 2022 (255 days post anti-CD5 CAR-T cell infusion), the proportion of CAR-T cells in CD3^+^ T cells was 1.92%, and no discernible lymph nodes were detected in neck and groin regions according to the (12)FDG-PET scan (**Supplemental Figure 3**). On August 30, 2023 (815 days post anti-CD5 CAR-T cell infusion), the CAR gene was still detectable in the peripheral blood, with a count of 1902 copies/μg DNA. These results suggest that the hyperplastic T cells observed in the inguinal lymph node are, in fact, expanded CAR-T cells rather than lymphoma cells.

## Discussion

ALK^+^ ALCL is among the most prevalent subtypes of T-cell lymphoma, accounting for 10-30% of all lymphomas in adolescent (11). There is a noted male predominance, and a substantial majority (50%-70%) of patients present with stage III-IV advanced disease (13, 14). As of the 2020 National Comprehensive Cancer Network (NCCN) guidelines, clinical trials were recommended as a primary treatment approach. While CAR-T cell immunotherapy has demonstrated high therapy efficacy in advanced B-cell malignancies (14), its application in T-cell malignancies requires further exploration and refinement. In this case study, we illustrate that anti-CD5 CAR-T cells exhibited enhanced and effective therapeutic potential in the treatment of chemotherapy-refractory ALK^+^ ALCL.

CD5 acts as an inhibitory regulator of the T cell receptor and has been considered a compelling target for CAR-T cell therapy, primarily due to its presence in approximately 85% of T cell malignancies (15). In our previous study, we optimized the production of anti-CD5 CAR-T cells, which demonstrated rapid and potent cytotoxicity against CD5-expressing tumor cell lines. Moreover, these anti-CD5 CAR-T cells effectively suppressed tumor growth in *in vivo* models derived from cell lines, known as cell line-derived xenograft (CDX) mice models (8).

The side effects of CAR-T cell therapy in T cell malignance have been documented (15–17). Among these, adverse events are a primary concern during the immunotherapy. Interventions such as steroids, corticosteroids, tocilizumab, dexamethasone, and ruxolitinib have been employed to mitigate CRS in the course of CAR-T treatment (12, 18, 19). In this specific case, the most severe adverse event manifested was a grade 4 rash on the skin. This manifestation may potentially be linked to the hyperplastic expansion of CAR-T cells, as the expansion can induce lymph nodes enlargement. Thus, it is imperative to engineer “live drug” CAR-T cells with a controllable switch, enabling clinicians to intervene in adverse effects. Suicide switches like inducible caspase 9 and the HSV-TK system have been explored in clinical trials and research (20, 21). In this study, a truncated epidermal growth factor receptor (EGFRt) was incorporated into the CAR structure (**Figure 1C**). This gene fragment was shown to be expressed on the CAR-T cell membrane, which can be recognized by Cetuximab and efficiently facilitates the elimination of CAR-T cells through NK cell dependent antibody-dependent cell cytotoxicity (ADCC) (22).

Following the administration of anti-CD5 CAR-T cell, there was a notable depletion of CD5 positive T cells, encompassing both malignant and normal T cells, observed in the patient’s peripheral blood (**Figure 1D**). Notably, no clinical manifestations or conditions associated with CD5^+^ T-cell deficiency were identified during the subsequent follow-up assessments. These findings align with results reported in analogous CD5 targeting clinical trials for T cell malignancies (15).

For various hematological diseases, establishing a sensitive and specific laboratory diagnosis system is of paramount importance, encompassing preliminary screening, confirmation experiments, and molecular biology assays. For example, in the case of leukemia, a morphology, immunology, cytogenetics, and molecular biology typing method is employed for accurate diagnosis. However, with the advent of new immunotherapeutic modalities such as monoclonal antibodies and CAR-T cell products, it is crucial to timely update laboratory diagnostic techniques to better inform precise treatment strategies. Novel technologies and methodology-driven assessments are progressively finding application in clinal therapy. In the presented case, the hyperplastic T lymphocytes observed in inguinal lymph node, exhibiting histopathological characteristics indicative of relapse or CAR-T cell lymphoma, were conclusively identified as CAR-T cells through the utilization of flow cytometry, gene rearrangement sequencing, and immune repertoire assays (**Figures 2C-E**). Hence, clinical practice must be intimately integrated with laboratory diagnosis to ensure precise clinical assessments.

## Data availability statement

The original contributions presented in the study are included in the article/**Supplementary Material**, further inquiries can be directed to the corresponding authors.

## Ethics statement

The studies involving humans were approved by Tongji Hospital, Tongji Medical College, Huazhong University of Science and Technology. The studies were conducted in accordance with the local legislation and institutional requirements. Written informed consent for participation in this study was provided by the participants’ legal guardians/next of kin. Written informed consent was obtained from the individual(s), and minor(s)’ legal guardian/next of kin, for the publication of any potentially identifiable images or data included in this article. Written informed consent was obtained from the participant/patient(s) for the publication of this case report.

## Author contributions

WM: Funding acquisition, Writing – original draft. MZ: Investigation, Writing – original draft. GH: Investigation, Project administration, Writing – original draft. YH: Methodology, Formal Analysis, Writing – original draft. XM: Formal Analysis, Methodology, Writing – original draft. CC: Methodology, Formal Analysis, Writing – original draft. KS: Methodology, Writing – original draft. ZD: Writing – review & editing. XJZ: Supervision, Writing – review & editing. XXZ: Supervision, Writing – review & editing. LH: Supervision, Writing – review & editing. QA: Supervision, Writing – review & editing. MX: Supervision, Funding acquisition, Writing – review & editing.
