# Supplementary material for: Case report: Differential diagnosis of highly amplified anti-CD5 CAR T cells and relapsed lymphoma cells in a patient with refractory ALK positive anaplastic large cell lymphoma
Source: Front Immunol. 2023 Dec 8;14:1280007. doi: 10.3389/fimmu.2023.1280007 (PMC10749197; doi:10.3389/fimmu.2023.1280007)
Supplement: Supplementary file 1 [file DataSheet_1.pdf]

Supplemental Figure 1

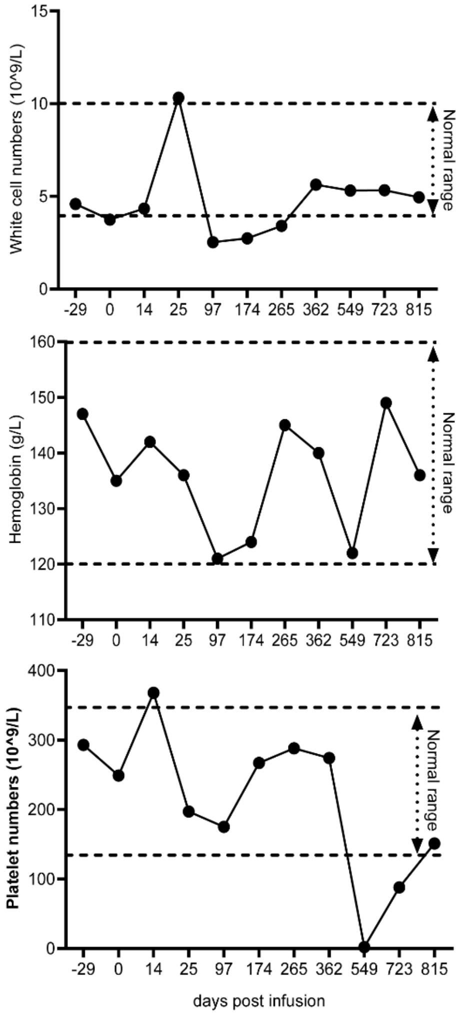

white blood cells number, platelets number and hemoglobin level post anti-CD5 CAR-T cells infusion. White cell numbers (upper), Hemoglobin (middle) and platelet numbers in patient after CAR-T cell treatment.

Supplemental Figure 2

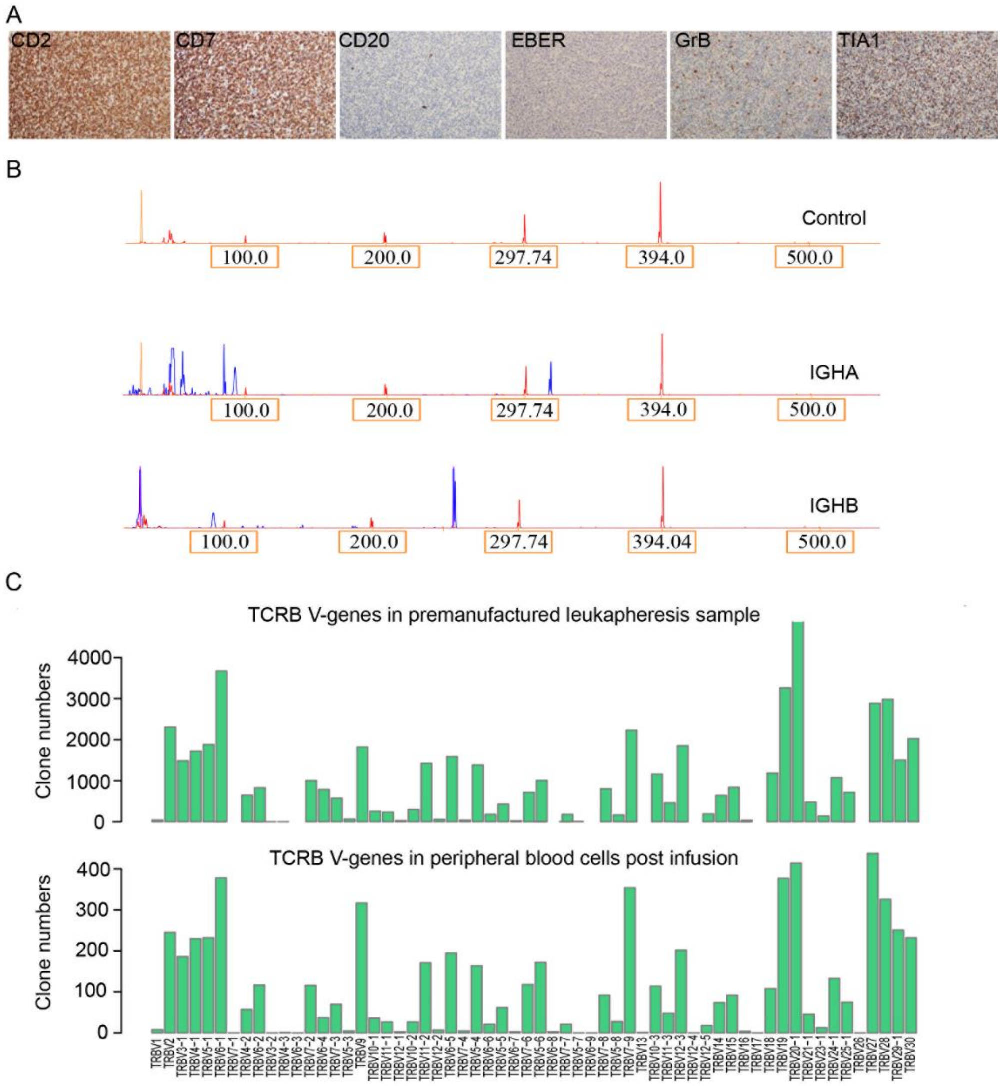

Characteristics of hyperplastic T cells. (A) Immunohistochemical analysis of cervical lymph node biopsy for CD2, CD7, CD20, EBER, GrB and TIA1 expression. (B) Rearrangement fragment of *IGHA* and *IGHB* verified by sanger sequencing. (C) Diversity of TCR clones in premanufactured leukapheresis sample (upper) and peripheral blood cells 68 days post infusion (lower). 53808 and 6474 clones were detected and the Shannon diversity is 14.47 and 8.97 respectively.

Supplemental Figure 3

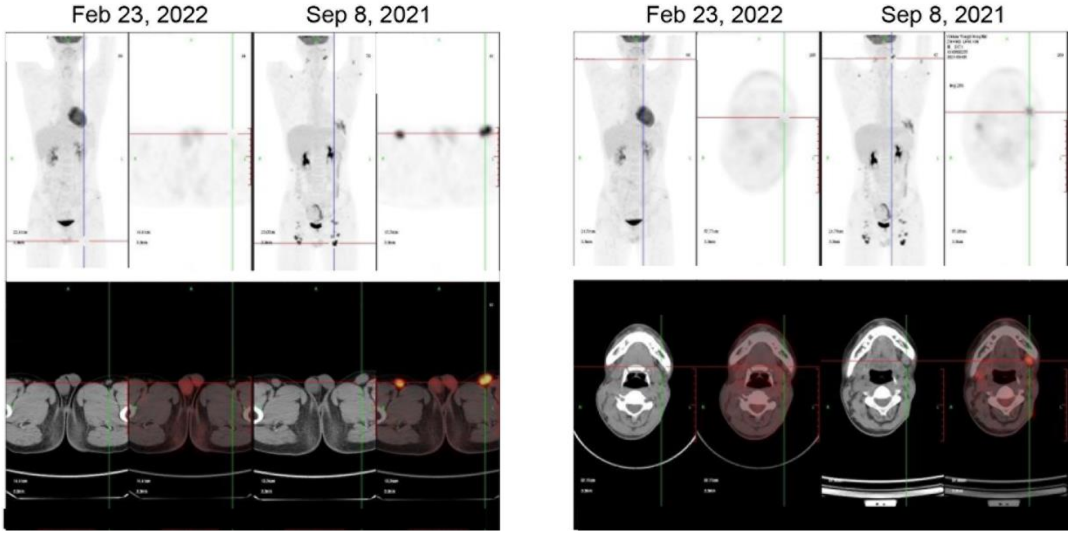

Inguinal lymph (left) and neck nodules (right) were scanned by ^{18}FDG-PET. No measurable nodes were found on Feb 23, 2022.
